# Supplementary material for: The genetic architecture of age-related hearing impairment revealed by genome-wide association analysis
Source: Commun Biol. 2021 Jun 9;4:706. doi: 10.1038/s42003-021-02224-9 (PMC8190123; doi:10.1038/s42003-021-02224-9)
Supplement: Supplementary file 2 — Supplementary Information [file 42003_2021_2224_MOESM2_ESM.pdf]

Supplementary Information

The genetic architecture of age-related hearing impairment revealed by genome-wide association analysis

Ivarsdottir er al.

SUPPLEMENTARY FIGURES

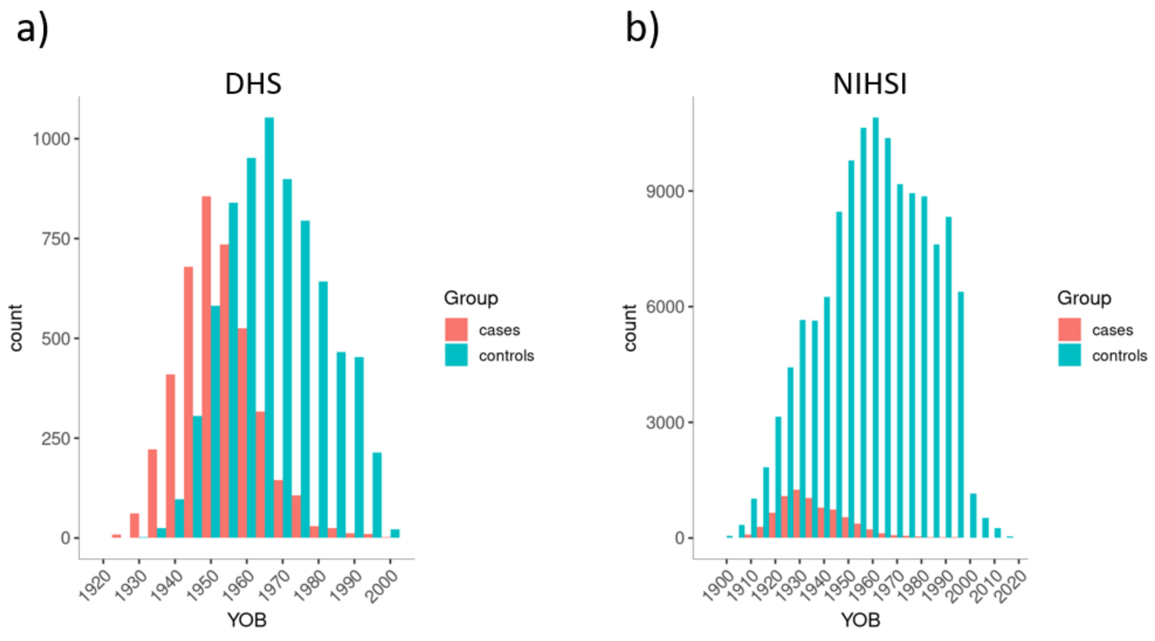

Supplementary Figure 1. The year of birth (YOB) distribution in a) the DHS dataset and b) NIHSI dataset. The orange bars show the YOB count for cases and the blue bars show the YOB count for controls.

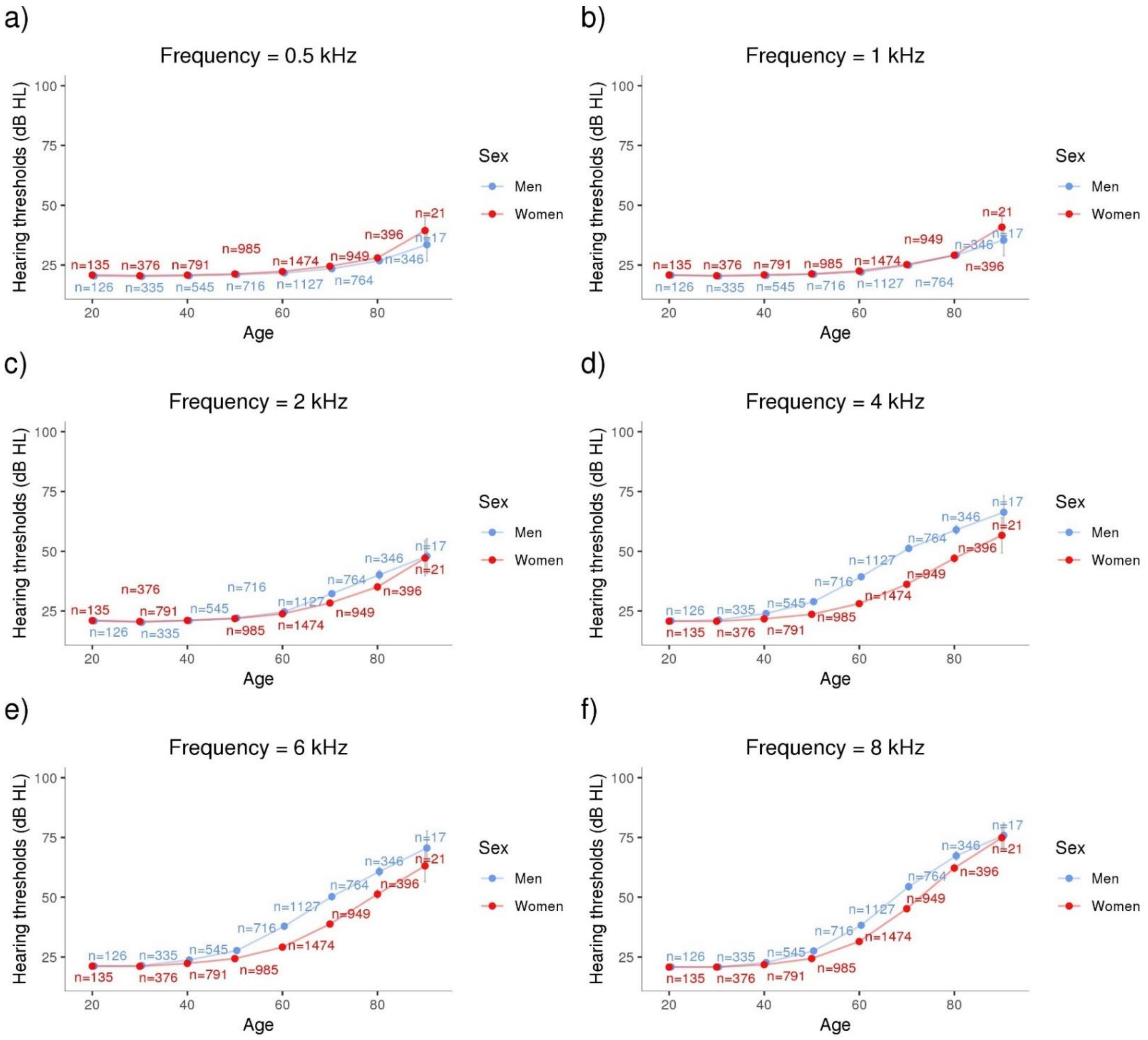

Supplementary Figure 2. Hearing thresholds per sex and age groups in the DHS dataset. Shown are the mean hearing thresholds per age group among men (blue dots) and women (red dots) for frequencies **a)** 0.5, **b)** 1, **c)** 2, **d)** 4, **e)** 6 and **f)** 8 kHz. The gray lines represent 95% confidence intervals.

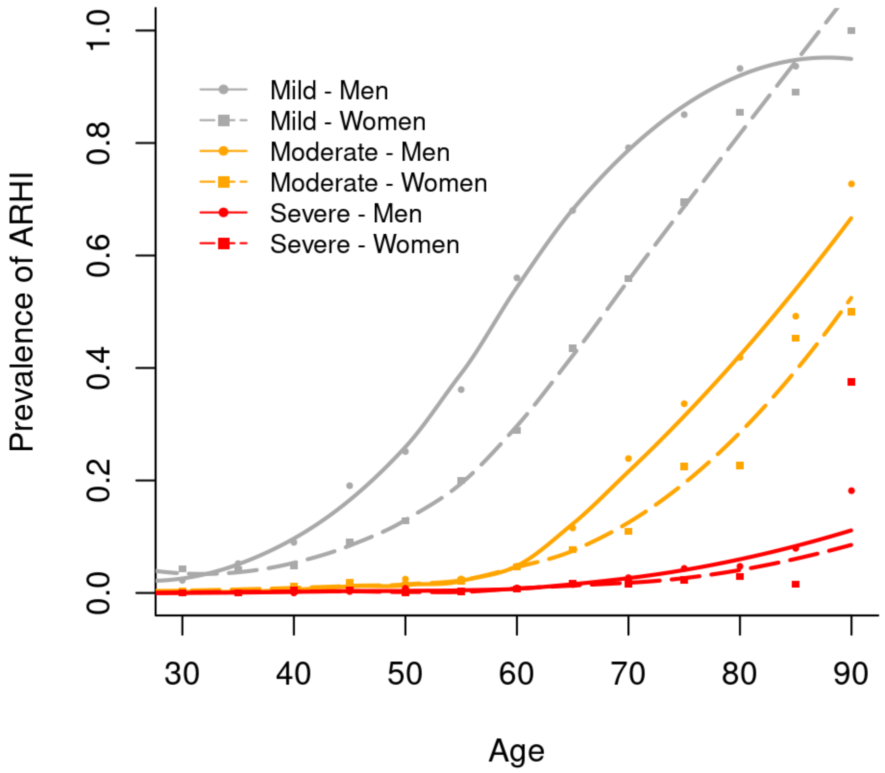

Supplementary Figure 3. The prevalence of ARHI vs age in the DHS dataset. Shown are the fractions of women (square dots) and men (round dots) per age group with mild in grey, moderate in yellow and severe hearing impairment in red.

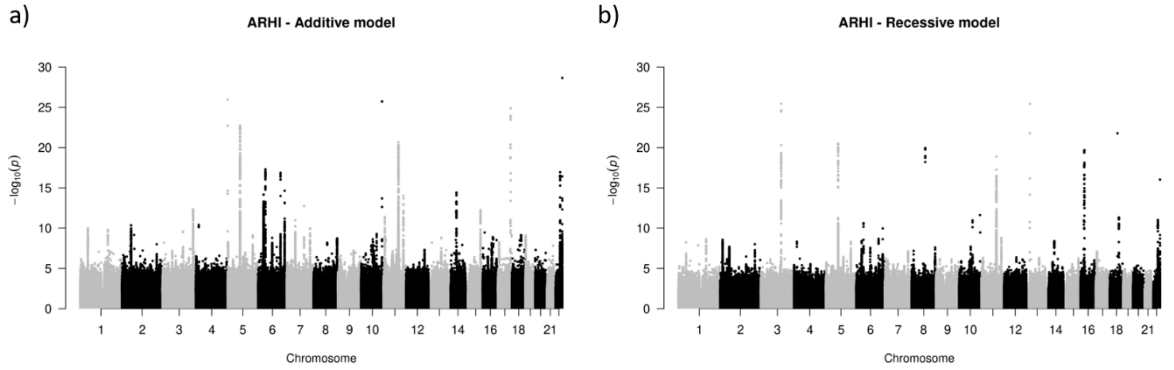

Supplementary Figure 4. Manhattan plots. Association results for the ARHI meta-analysis for **a)** the additive model and **b)** the recessive model. The −log10 P-values are plotted for each variant against their chromosomal position. A likelihood-ratio test was used when testing for association.

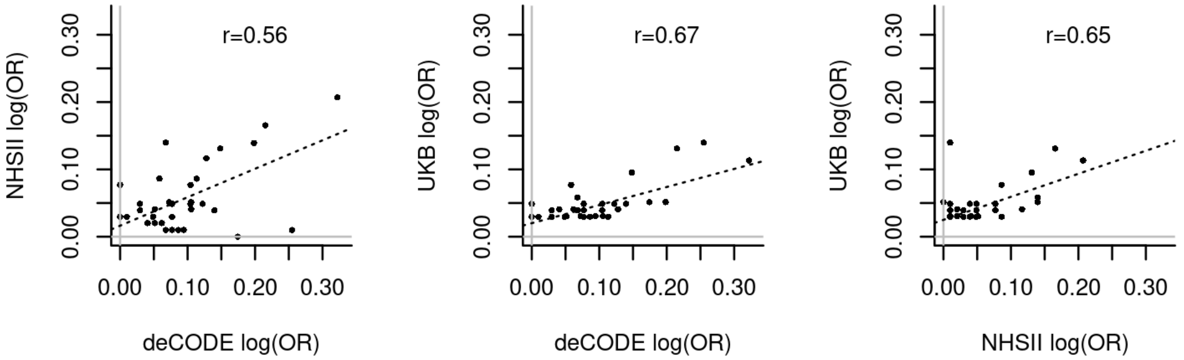

Supplementary Figure 5. Correlation between effect sizes obtained from the three GWAS datasets. Shown are the effect sizes for the 35 common ARHI variants (EAF<1%). The dotted lines represent results from weighted linear regression using MAF(1-MAF) as weights. The weighted correlation coefficients (r) are shown in the figures.

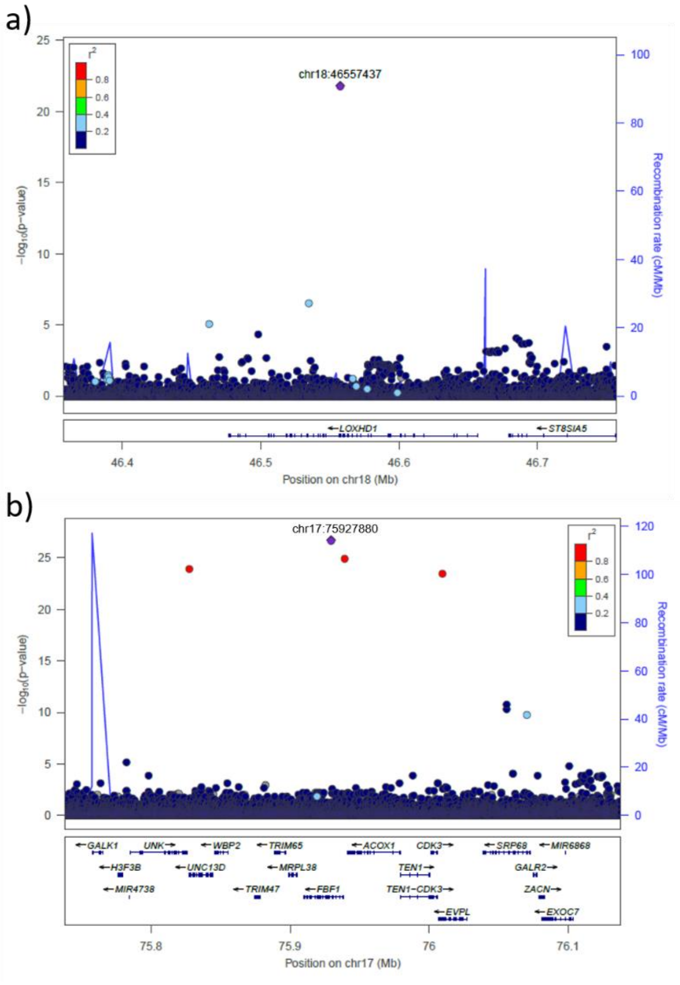

Supplementary Figure 6. Locus-plots showing variants at the **a)** *LOXHD1* and **b)** *FBF1* loci (hg38) associating with ARHI. The top variants are labelled as purple diamonds, other variants are colored according to correlation (r^2) with the top variants. $-\log_{10}$ P-values are shown on the left y-axis and the right y-axis shows calculated recombination rates at the chromosomal location, plotted as a blue line.

SUPPLEMENTARY TABLES

Supplementary Table 1. Hearing thresholds.

Hearing threshold (dB HL)	Hearing impairment
26-40	Mild
41-60	Moderate
61-80	Severe
≥81	Profound

Supplementary Table 2. Meta-analysis.

Datasets	Data	N cases	N controls
deCODE health study (DHS)	Air conduction audiometric test	4,140	7,344
National Institute of Hearing and Speech in Iceland (NIHSI)	Air conduction audiometric test	9,619	298,609
UK Biobank (UKB)	Self-reported hearing difficulty	108,175	285,746
		121,934	591,699

Supplementary Table 3. Carriers of potentially high impact mutations in the DHS. Homozygous or compound heterozygous carriers of rare LOF variants and heterozygous carriers of rare LOF variants in constrained genes were invited to participate in DHS. The table shows the number of carriers with mutations in Mendelian deafness genes (Hereditary Hearing Loss homepage, URLs) that were invited and how many of them participated. The table also shows the number of homozygous carriers of p.Arg1090Gln in *LOXHD1* that were additionally recruited after discovering the association between p.Arg1090Gln and ARHI.

	N invited	N participated
Carriers of p.Arg1090Gln in *LOXHD1*	40	20
Homozygous or compound heterozygous carriers of rare LOF variants in Mendelian deafness genes (*GJB2, GJB6, LRTOMT, OTOF, PCDH15, PPIP5K2, SLC26A5, TMPRSS3, TNC, TRIOBP* and *TSPEAR*)	81	26
Heterozygous carriers of rare LOF variants in constrained Mendelian deafness genes (*ADCY1, COL11A2, DMXL2, MYH14, MYH9, RDX* and *TRRAP*)	50	23
Total	171	69

Supplementary Table 4. Summary of audiometric measures from the NIHSI dataset (N=14,329). For each frequency, the mean, standard deviation (SD) and range of the hearing thresholds is shown. The prevalence of mild, moderate, severe and profound hearing impairment is shown for each frequency. The effect of age in SD, sex given for women and height in SD on ARHI (PTA>25 dB HL) and the corresponding P-values are shown for each frequency.

				**Hearing impairment prevalence (%)**				**Effect of age and sex on ARHI**				**Effect of age, sex and height on ARHI**					
**kHz**	**Mean**	**SD**	**Range**	**Mild**	**Moderate**	**Severe**	**Profound**	**Age effect**	**P-value**	**Sex effect**	**P-value**	**Age effect**	**P-value**	**Sex effect**	**P-value**	**Height effect**	**P-value**
0.5	28.3	19.7	-8;120	46.6%	23.7%	6.3%	1.7%	2.88	$<1\times10^{-300}$	1.85	1.7×10^{-60}	3.06	$<1\times10^{-300}$	1.39	2.4×10^{-7}	0.79	6.7×10^{-12}
1	33.9	21.4	-8;120	58.7%	34.7%	10.1%	2.6%	3.81	$<1\times10^{-300}$	1.79	1.1×10^{-48}	4.13	$<1\times10^{-300}$	1.33	2.5×10^{-5}	0.79	2.3×10^{-11}
2	41.3	23.1	-8;120	71.3%	51.9%	17.8%	4.0%	6.02	$<1\times10^{-300}$	1.24	3.2×10^{-6}	6.62	$<1\times10^{-300}$	0.89	0.15	0.80	3.4×10^{-8}
4	54.1	24.4	-8;120	83.3%	72.0%	42.8%	10.9%	9.68	$<1\times10^{-300}$	0.39	2.7×10^{-58}	10.76	$<1\times10^{-300}$	0.26	1.3×10^{-37}	0.82	2.8×10^{-4}
6	58.1	26.0	-10;120	83.8%	74.1%	51.8%	17.2%	7.11	$<1\times10^{-300}$	0.69	1.2×10^{-11}	7.72	$<1\times10^{-300}$	0.61	2.5×10^{-7}	0.95	0.33
8	60.2	27.3	-10;120	83.2%	75.4%	56.9%	22.9%	9.65	$<1\times10^{-300}$	0.68	1.8×10^{-11}	10.62	$<1\times10^{-300}$	0.49	3.5×10^{-12}	0.83	4.6×10^{-4}

kHz = kilohertz

Supplementary Table 5. Significance thresholds for each variant category. The thresholds were estimated from the Icelandic data as previously described[1].

Category	Variants in category	Broad DHS	Significance threshold
High impact	Splice donor variant, splice acceptor variant, stop gained, frameshift variant, stop lost, initiator codon variant.	-	2.4×10^{-7}
Moderate impact	Inframe indels, missense, splice region variants, stop retained variants.	-	4.9×10^{-8}
Low impact	Synonymous variants, 5' UTR variant, 3' UTR variant, up- and downstream variants, coding sequence variants.	-	4.4×10^{-9}
Other	Intronic, intergenic variants.	Yes	2.2×10^{-9}
		No	7.7×10^{-10}

DHS = Dnase I hypersensitivity sites

Supplementary Table 6. The differential expression of ARHI genes in hair cells versus non-hair cells in mice. Data was obtained from the SHIELD database[2,3]. The GFP+ cells include hair cells from the cochlea and the utricle and the table also shows the fold change for Cochlea/Utricle expression.

Gene	Fold Change GFP+/GFP-	FDR	Fold Change Cochlea/Utricle	FDR
SLC26A5	171.13	1.0×10^{-9}	147.44	1.4×10^{-4}
CRIP3	55.36	3.1×10^{-6}	0.17	0.058
FSCN2	42.83	2.5×10^{-7}	0.61	1
TMPRSS3	42.33	1.9×10^{-9}	1.75	1
CDH23	32.06	1.4×10^{-2}	1.01	1
CCDC68	23.30	1.8×10^{-7}	0.11	0.26
C10orf90	17.69	1.3×10^{-4}	1.01	1
SNAP91	15.89	3.2×10^{-6}	0.72	1
SH2D4B	15.23	5.1×10^{-2}	1.61	1
LMO7	13.94	5.0×10^{-6}	0.83	1
ABLIM3	7.95	2.9×10^{-3}	1.18	1
ILDR1	7.65	1.3×10^{-4}	1.07	1
EYA4	7.55	6.1×10^{-3}	0.68	1
ATP6V0A4	7.24	0.13	1.35	1
AP1M2	6.88	3.4×10^{-3}	1.28	1
SYNJ2	6.88	1.0×10^{-2}	0.74	1
TUB	5.91	3.8×10^{-2}	5.42	0.87
TRIOBP	4.56	8.6×10^{-2}	1.11	1
TBX2	4.55	0.36	0.33	0.87
BAIAP2L2	4.17	9.9×10^{-2}	1.98	1
ARHGEF28	3.19	6.5×10^{-2}	1.06	1
EXOC6	3.08	0.44	1.17	1
CHMP4C	2.82	0.45	0.39	1
CTBP2	2.24	0.45	0.74	1
CCDC17	1.73	0.86	0.95	1
WDR3	1.28	0.73	1.44	1
ABCC10	1.17	0.95	0.38	1
MPZL2	1.00	0.92	0.61	1
AGO2	0.93	1.0	1.00	1
FBF1	0.76	0.75	0.75	1
TBC1D24	0.75	1.0	1.31	1
SPTBN1	0.72	0.99	1.18	1
SLC4A11	0.65	0.71	0.47	1
ACADVL	0.61	0.85	0.74	1
TYR	0.53	0.58	1.27	1
GRB10	0.44	0.50	1.63	1
HLA-DRB1	0.36	0.56	0.46	1
LMX1A	0.27	0.13	0.57	0.38
PHLDB1	0.24	0.34	0.46	1
TMPRSS9	0.24	0.39	17.97	0.46
ISG20	0.17	0.15	0.21	0.35
NID2	0.08	7.1×10^{-3}	0.47	0.53
FLJ46066	-	-	-	-
CLRN2	-	-	-	-
KLHDC7B	-	-	-	-
LOXHD1	-	-	-	-

FDR=False discovery rate

Supplementary Table 7. The association of ARHI variants with tinnitus. Logistic regression was used to test the association between self-reported tinnitus and sequence variants in the DHS and UKB datasets. The effects (OR) from meta-analyzing the two datasets are given for the minor allele. Effects are marked with a * for significant association after controlling the false discovery rate at 0.05 with the Benjamini-Hochberg procedure. For intergenic variants, the nearest gene is reported in brackets. ARHI variants detected under the additive model were tested for tinnitus using the additive model and ARHI variants detected under the recessive model were tested for tinnitus using the recessive model. This is the data underlying Figure 6.

					**Tinnitus**	
**Model**	**Chrom**	**Position**	**rs name**	**Gene**	**P-value**	**OR**
A	10	125123701	rs10901863	*CTBP2*	4.9×10^{-6}	1.04*
A	6	43305866	rs2242416	*CRIP3*	4.3×10^{-5}	0.97*
R	3	121993204	rs2877561	*ILDR1*	1.8×10^{-4}	1.08*
A	8	140637192	rs11996715	*AGO2*	5.3×10^{-4}	1.03*
R	6	43433367	rs9394952	*ABCC10*	1.7×10^{-3}	1.04*
A	11	118689022	rs113784020	*[PHLDB1]*	2.4×10^{-3}	1.06*
A	1	165139894	rs7525101	*[LMX1A]*	2.9×10^{-3}	1.02*
A	7	103421378	rs141952919	*SLC26A5*	6.0×10^{-3}	1.15*
A	17	7224399	rs17671352	*ACADVL*	6.6×10^{-3}	1.02*
R	10	80649861	rs12784122	*SH2D4B*	7.4×10^{-3}	1.82*
A	6	158071628	rs146694394	*SYNJ2*	8.8×10^{-3}	1.17*
R	10	126459169	rs139123090	*C10orf90*	1.0×10^{-2}	9.24*
A	4	17522947	rs13147559	*CLRN2*	1.2×10^{-2}	1.03*
A	10	71617355	rs143282422	*CDH23*	1.7×10^{-2}	1.09
A	19	2389142	rs11881070	*TMPRSS9*	1.9×10^{-2}	0.98
A	5	73780686	rs6453022	*ARHGEF28*	3.1×10^{-2}	1.02
A	7	50785454	rs11238325	*GRB10*	3.4×10^{-2}	0.98
A	22	50549067	rs749405486	*KLHDC7B*	5.7×10^{-2}	1.36
A	6	83607814	rs7752421	*SNAP91*	5.9×10^{-2}	1.02
A	14	52048194	rs1566129	*NID2*	6.5×10^{-2}	1.01
A	11	118609508	rs67307131	*PHLDB1*	6.7×10^{-2}	1.02
A	2	54590546	rs6545432	*SPTBN1*	0.10	0.99
R	8	81753241	rs137960856	*CHMP4C*	0.10	1.14
A	11	89284793	rs1126809	*TYR*	0.10	1.01
R	18	46557437	rs118174674	*LOXHD1*	0.12	1.43
A	22	50549676	rs36062310	*KLHDC7B*	0.12	1.03
A	6	133468590	rs9493627	*EYA4*	0.14	1.01
A	16	2497068	rs761934676	*TBC1D24*	0.16	1.61
R	18	54937957	rs1344011	*CCDC68*	0.16	1.04
A	6	32593597	rs201291779	*HLA-DRB1*	0.17	1.04
A	5	149221680	rs13171669	*ABLIM3*	0.18	1.01
A	3	182320787	rs73189920	*[FLJ46066]*	0.19	1.02
A	5	73626822	rs2703636	*ARHGEF28*	0.20	1.01
A	3	182285702	rs72622588	*[FLJ46066]*	0.22	0.99
R	22	37735350	rs200045032	*TRIOBP*	0.22	3.66
A	7	138802400	rs2354376	*ATP6V0A4*	0.23	0.99
A	22	37726115	rs5756795	*TRIOBP*	0.27	1.01
R	1	117960109	rs557563970	*WDR3*	0.30	0.98
A	17	81528943	rs143796236	*FSCN2*	0.32	1.05
A	13	75842965	rs920701	*LMO7*	0.37	0.99
A	15	88693155	rs12441297	*[ISG20]*	0.40	0.99
A	21	42389042	rs727503493	*TMPRSS3*	0.43	1.16
A	10	93023116	rs10531957	*EXOC6*	0.44	0.99
A	20	3228565	rs764272881	*SLC4A11*	0.62	0.87
A	11	8035366	rs55635402	*TUB*	0.74	1.00
A	6	158086949	rs2296506	*SYNJ2*	0.82	1.00
A	22	38087148	rs132924	*BAIAP2L2*	0.82	1.00
A	17	75927880	-	*FBF1*	0.89	1.06
A	1	45620405	rs3014246	*CCDC17*	0.93	1.00
R	11	118262596	rs74543584	*MPZL2*	0.93	1.06

A=Additive model, R=Recessive model

SUPPLEMENTARY NOTES

Supplementary Note 1

In the meta-analysis a rare intronic variant in *EXOC6B* satisfied the genome-wide significance threshold. The association was driven by a single family where 6 carriers showed mild to profound hearing impairment in the NIHSI dataset. We whole-genome sequenced (WGS) 4 of the individuals and found that all of them are carriers of a stop-gained variant, p.Tyr266Ter, in *TBX2* that had been excluded in the original GWAS because of low imputation info. A microdeletion in 17q23.2, which includes *TBX2*, has been reported to cause sensorineural hearing loss among other clinical features[6]. Out of 50K WGS individuals, the p.Tyr266Ter variant was only present in descendants of a man born in 1900, who we call subject 1. We performed Sanger sequencing of p.Tyr266Ter in all descendants of subject 1 with a blood sample available (N=17), and in all individuals that had genotype probability >0 (N=15) and an available sample. In total 10 descendants of subject 1 are confirmed carriers of p.Tyr266Ter, 8 of whom have confirmed mild to profound hearing impairment. Two additional descendants have hearing impairment but their samples were not available for genotype assessment. After obtaining these results, we tested the variant again for association with ARHI (OR= 71.2, $P=4.1\times10^{-8}$), and conclude that p.Tyr266Ter in *TBX2* is the cause of hearing impairment in this family. Furthermore, we found another predicted loss-of-function variant in *TBX2*, p.Glu309SerfsTer59, in an individual unrelated to this family, who shows mild hearing impairment at 14 years of age.

Supplementary Note 2

32 bp sequence unique to exon 7 to exon 4 splicing (reverse compliment of cDNA) in ENST00000586717.5

```
TTCTCAGGTAGTGTCACTGCAGGCTTGGGGTG
   exon 4           exon 7
```

32 bp sequence unique to exon 7 to exon 8 (reverse compliment of cDNA) in ENST00000586717.5

```
GTGGGAATGGCACCCCCTGCAGGCTTGGGGTG
   exon 8           exon 7
```

The exon 7 (ENSE00003798535) splicing usage served as a proxy for *FBF1* transcripts expression with and without duplication, where the exon 7 to exon 8 junction is contained in all *FBF1* transcripts and the exon 7 to exon 4 junction in duplicated transcripts. Percentage of exon 7 to 4 duplication usage was estimated from normal splice counts (nCount) and duplication splice counts (dCounts) retrieved from RNA-sequencing data of 55 carriers with minimal *FBF1* expression. The slope coefficient of generalized linear model in R, dCount ~ 0 + nCount, was computed to estimate the duplication usage, assuming binomial distribution of counts.

Supplementary Note 3

The missense variant in *MPZL2*, p.Asp93Val, associates with ARHI under the recessive model (MAF_{UK}=0.83%, $MAF_{Iceland}$=1.47%, OR=4.0, P=2.0×10^{-7}). Two recent studies report biallelic loss-of-function variants in *MPZL2* as a cause of early-onset, mild to profound hearing loss and that Mpzl2 is expressed in mouse inner ear[7,8] (DFNB111, OMIM #618145). In Iceland, eight homozygous carriers of p.Asp93Val in *MPZL2* had audiometric data, seven of whom showed mild to severe ARHI (Figure 4.c).

The missense variant in *SLC4A11*, p.Ser779Pro, associates with ARHI under the additive model (OR=1.81, P=4.9×10^{-8}) (Figure 4.d). The variant has MAF=0.45% in Iceland but was not present in the UK Biobank data. Various quality measures are calculated for imputed variants as a part of quality filtering, among them are *phasing* r^2 and *leave-one-out* r^2. *Phasing* r^2 is defined as r^2 between phased genotypes and observed genotypes from sequencing, and *leave-one-out* r^2 is defined as r^2 between the observed genotypes from sequencing and genotypes imputed into each sequenced individual from all the other sequenced individuals. The variant, p.Ser779Pro, was flagged in quality filtering due to *phasing* r^2 and *leave-one-out* r^2 being less than 0.8. This was mainly due to 136 sequenced carriers losing their carrier status in phasing. The phasing program takes haplotype sharing into account and can change the genotype of an expected carrier if the genotype is not completely clear in the WGS data and the haplotype sharing with other samples indicates a different genotype. To investigate this, we Sanger sequenced 132 of the 136 individuals where the sequenced genotype did not match the phased genotype, as well as 56 carriers with ARHI and found that the Sanger sequencing matched the phased genotypes

for all 188 individuals. These results show that the phasing of the variant is correct and this quality flag does not affect the association results. Mutations in *SLC4A11* are known to cause corneal endothelial dystrophy, dominant and recessive forms, as well as Harboyan syndrome, which consists of congenital corneal dystrophy and progressive postlingual sensorineural hearing loss (OMIM #217400). It is not known why some mutations cause corneal endothelial dystrophy with hearing loss and some without hearing loss. In the deCODE health study, several ocular traits were obtained[9]. Interestingly, p.Ser779Pro associates strongly with endothelial cell density (β=-0.70 (SD), P=1.4×10^{-9}), corneal hysteresis (β=-0.83 (SD), P=1.1×10^{-12}) and central corneal thickness (β=-0.64 (SD), P=3.3×10^{-8}), but does not associate with increased risk of hereditary corneal dystrophies (ICD code H185) (OR=1.13, P=0.91).

The missense variant in *SLC26A5*, p.Leu46Pro, associates with increased risk of ARHI (MAF_{UK}=0.52%, $MAF_{Iceland}$=0.33%, OR=1.28, P=1.8×10^{-13}) under the additive model (Figure 2.e). *SLC26A5* encodes prestin, which functions as a molecular motor in the outer hair cells of the cochlea and mediates sound amplification[10,11]. Rare LOF variants in *SLC26A5* have been reported to cause autosomal recessive deafness (DFNB61, OMIM #613865).

Using the WES UK Biobank data, we detected a rare missense variant, p.Asn307Ser, in *TBC1D24*, associating with increased risk of ARHI under the additive model (MAF_{UK}=0.01%, OR=1.28, P=1.8×10^{-13}). The variant is not present in Iceland. Other variants in *TBC1D24* have been associated with several clinical symptoms including both dominant and recessive form of non-syndromic deafness (DFNA65, OMIM #616044 and DFNB86, OMIM #614617), epilepsy and DOORS syndrome which is characterized by deafness, onychodystrophy, osteodystrophy, intellectual disability, and seizures (OMIM #220500). Seventy-one carriers of the variants answered the hearing questions in UK Biobank, 45 of whom said they had difficulty with hearing, 18 reported the use of hearing aids but none of them stated that they were completely deaf. This suggests that p.Asn307Ser, causes ARHI or a milder form of hearing loss than previously described pathogenic variants in *TBC1D24*.

Two variants in *TRIOBP* associate independently with ARHI. One is a previously reported ARHI signal[12] represented by a common missense variant, p.Phe1187Leu, that associates with ARHI under an additive model ($MAF_{Iceland}$=45.1%, MAF_{UK}=46.4%, OR=1.04, P=1.1×10^{-17}). The other is a low-frequency variant, p.Gly1672Ter, that is predicted to result in a premature termination of translation and associates with increased risk of ARHI under a recessive model in the Icelandic data ($MAF_{Iceland}$ = 0.75%, OR=69.6, P=2.7×10^{-7}) (Table 2). Homozygous mutations in *TRIOBP* are known to cause autosomal recessive deafness (DFNB28, OMIM #609823) characterized by prelingual, severe to profound sensorineural hearing loss. We do not list p.Gly1672Ter as one of the novel variants, since two recent studies have reported p.Gly1672Ter in compound heterozygosity, causing milder hearing loss than other described *TRIOBP* mutations[13,14]. However, it has not been reported in homozygosity before. p.Gly1672Ter is less frequent in the UK (MAF_{UK}=0.04%) and other European populations (MAF=0.08%) (gnomAD URLs) and no homozygous carriers are present in the UK Biobank data set. Due to the recruitment procedure in the deCODE health study (enriched for carriers of rare and potentially high impact variants), five homozygous carriers of p.Gly1672Ter, aged between 31 and 80 years old, were recruited, all of whom showed mild or moderate ARHI. *TRIOBP* isoforms can be grouped into three classes; TRIOBP-1, TRIOBP-4 and TRIOBP-5[15]. All pathogenic mutations associated with DEFNB28 only affect TRIOBP-4 or TRIOBP-5, which are specifically expressed in the inner ear and retina of both humans and mice[14,15]. p.Gly1672Ter is the only pathogenic variant that exclusively affects the TRIOBP-5 isoform[13], and previous studies reported it in compound heterozygosity with variants that affect TRIOBP-4[13,14]. Therefore, our finding demonstrates the first *TRIOBP* mutation only affecting TRIOBP-5 that associates with hearing impairment.

SUPPLEMENTARY REFERENCES

1. Sveinbjornsson, G. *et al.* Weighting sequence variants based on their annotation increases power of whole-genome association studies. *Nat. Genet.* **48,** 314–317 (2016).

2. Shen, J., Scheffer, D. I., Kwan, K. Y. & Corey, D. P. SHIELD: An integrative gene expression database for inner ear research. *Database* **2015,** (2015).

3. Scheffer, D. I., Shen, J., Corey, D. P. & Chen, Z.-Y. Gene Expression by Mouse Inner Ear Hair Cells during Development. *J. Neurosci.* **35,** 6366–6380 (2015).

4. Lucotte, G. & Diéterlen, F. The 35delG Mutation in the Connexin 26 Gene (GJB2) Associated with Congenital Deafness: European Carrier Frequencies and Evidence for Its Origin in Ancient Greece . *Genet. Test.* **9,** 20–25 (2005).

5. Lameiras, A. R. *et al.* The controversial p.Met34Thr variant in GJB2 gene: Two siblings, one genotype, two phenotypes. *Int. J. Pediatr. Otorhinolaryngol.* **79,** 1316–1319 (2015).

6. Nimmakayalu, M. *et al.* Microdeletion of 17q22q23.2 encompassing TBX2 and TBX4 in a patient with congenital microcephaly, thyroid duct cyst, sensorineural hearing loss, and pulmonary hypertension. *Am. J. Med. Genet. Part A* **155,** 418–423 (2011).

7. Wesdorp, M. *et al.* MPZL2, Encoding the Epithelial Junctional Protein Myelin Protein Zero-like 2, Is Essential for Hearing in Man and Mouse. *Am. J. Hum. Genet.* **103,** 74–88 (2018).

8. Bademci, G. *et al.* MPZL2 is a novel gene associated with autosomal recessive nonsyndromic moderate hearing loss. *Hum. Genet.* **137,** 479–486 (2018).

9. Ivarsdottir, E. V. *et al.* Sequence variation at ANAPC1 accounts for 24% of the variability in corneal endothelial cell density. *Nat. Commun.* **10,** (2019).

10. Zheng, J. *et al.* Prestin is the motor protein of cochlear outer hair cells. *Nature* **405,** 149–155

(2000).

11. Liu, X. Z. *et al.* Prestin, a cochlear motor protein, is defective in non-syndromic hearing loss. *Hum. Mol. Genet.* **12,** 1155–62 (2003).

12. Hoffmann, T. J. *et al.* A Large Genome-Wide Association Study of Age-Related Hearing Impairment Using Electronic Health Records. *PLoS Genet.* **12,** (2016).

13. Wesdorp, M. *et al.* Broadening the phenotype of DFNB28: Mutations in TRIOBP are associated with moderate, stable hereditary hearing impairment. *Hear. Res.* **347,** 56–62 (2017).

14. Pollak, A. *et al.* Whole exome sequencing identifies TRIOBP pathogenic variants as a cause of post-lingual bilateral moderate-to-severe sensorineural hearing loss. *BMC Med. Genet.* **18,** 1–9 (2017).

15. Kitajiri, S. *et al.* Actin-Bundling Protein TRIOBP Forms Resilient Rootlets of Hair Cell Stereocilia That Are Essential for Hearing. *Cell* **141,** 786–798 (2011).
