# Supplementary material for: The genetic architecture of age-related hearing impairment revealed by genome-wide association analysis
Source: Commun Biol. 2021 Jun 9;4:706. doi: 10.1038/s42003-021-02224-9 (PMC8190123; doi:10.1038/s42003-021-02224-9)
Supplement: Supplementary file 3 — Description of Supplementary Files [file 42003_2021_2224_MOESM3_ESM.pdf]

Description of Additional Supplementary Files

File name: Supplementary Data

Description:

Supplementary Data 1

Association of sequence variants with ARHI. The table shows the meta-analysis results for all ARHI associating variant, relevant information about the variants as well as the GWAS results in each dataset; DHS, NIHSI and UKB. The last column notes which variants were detected in meta-analysis II (see Figure 1 and methods) using the whole exome sequenced UKB dataset. The last column also notes the 6 loci where the previously reported variants are non-coding but we identified missense or splice region variants (r^2>0.85 between our top variant and the reported variant). For intergenic variants, the nearest genes are reported in brackets.

Supplementary Data 2

Association of sequence variants with childhood-onset hearing loss. The table shows the meta-analysis results for the variants that satisfied the genome-wide significant thresholds and were estimated to cause prelingual or childhood-onset hearing loss instead of ARHI.

Supplementary Data 3

The effect of ARHI variants on PTA based ARHI and ARHI at the frequencies 0.5, 1, 2, 4, 6 and 8 kHz. The table shows the results from a meta-analysis from the Icelandic datasets with audiometric measurements (DHS and NIHSI datasets). This is the data underlying Figure 5.

Supplementary Data 4

The effect of ARHI variants on ARHI per genotype. The table shows the effect of heterozygous and homozygous carriers on ARHI in each dataset, for variants where homozygous carriers exist. The deviation columns for each datasets shows the p-value when comparing the full genotypic model to the additive model using a likelihood ratio test. The last column shows the deviation meta-analysis p-value that was computed using sample size approach based on the deviation P-values and sample sizes from each dataset.

Supplementary Data 5

Association of sequence variants with ARHI in the DHS and NIHSI datasets using only individuals at least 40 years old. The table shows the results for all ARHI associating variant and relevant information about the variants. For intergenic variants, the nearest genes are reported in brackets.

Supplementary Data 6

Source data underlying Figure 2.a

Supplementary Data 7

Source data underlying Figure 2.b

Supplementary Data 8

Source data underlying Figure 2.c

Supplementary Data 9

Source data underlying Figure 3.a

Supplementary Data 10

Source data underlying Figure 4.a

Supplementary Data 11

Source data underlying Figure 4.b

Supplementary Data 12

Source data underlying Figure 4.c

Supplementary Data 13

Source data underlying Figure 4.d

Supplementary Data 14

Source data underlying Figure 4.e
